# Quiet Moment around the Campfire

**DOI:** 10.3201/eid2006.AC2006

**Published:** 2014-06

**Authors:** Byron Breedlove

**Affiliations:** Centers for Disease Control and Prevention, Atlanta, Georgia, USA

**Keywords:** art science connection, emerging infectious diseases, art and medicine, Frederic Remington, cowboys, The Cigarette, Quiet Moment around the Campfire, fungi, bats, rodents, coccidioidomycosis, measles, tuberculosis, pneumonia, typhoid, nocturnes, about the cover

**Figure:**
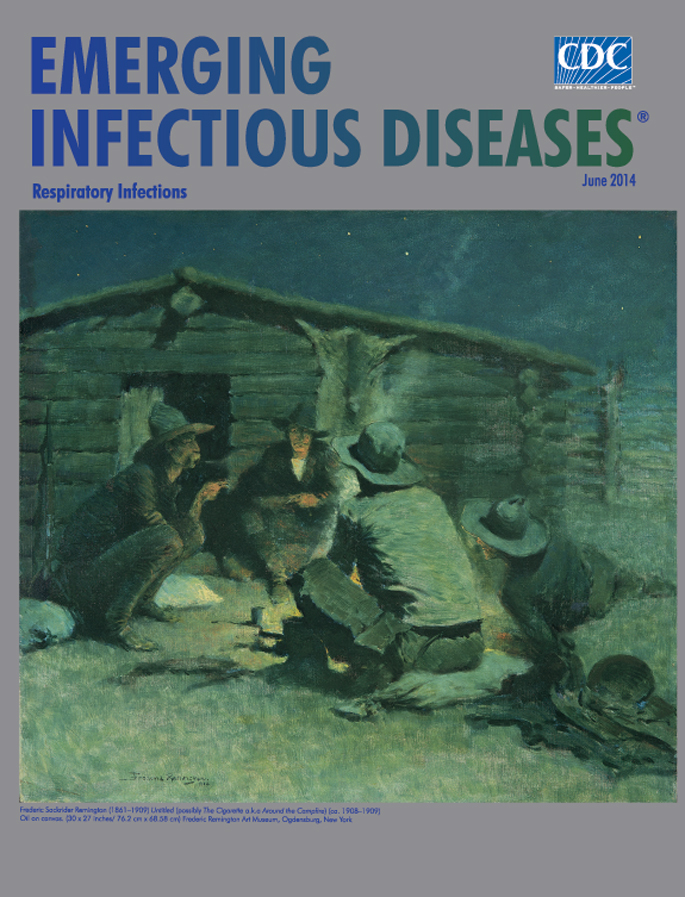
**Frederic Sackrider Remington (1861–1909) Untitled (possibly *The Cigarette* a.k.a. *Around the Campfire*) (ca. 1908–1909) Oil on canvas. (30 in × 27 in/76.2 cm × 68.58 cm)** Frederic Remington Art Museum, Ogdensburg, New York

Frederic Remington was an American painter, sculptor, illustrator, and writer whose works frequently featured cowboys, Native Americans, soldiers, horses, bison, and other iconic features of the rapidly vanishing American West. During his 25-year career, Remington produced ≈3,000 paintings and drawings, 22 bronze sculptures, a novel, a Broadway play, and more than 100 articles and stories. Remington was 48 years old when he died of peritonitis, a complication of an emergency appendectomy.

In approximately 1900, Remington began working on a series of paintings—now known collectively as the nocturnes—that depict color and light unique to night. Nancy Anderson, curator of American and British Paintings at the National Gallery of Art, Washington, DC, wrote that “In these experimental, complex, and deeply personal paintings, Remington explored the technical and aesthetic difficulties of painting darkness. Surprisingly, his images are filled with color and light—moonlight, firelight, candlelight.” Many of those paintings also depict danger from humans, animals, or nature, as implied and real threats, sometimes revealed to the viewer and sometimes suggested by the posture or action of Remington’s subjects.

This month’s cover painting, commonly known as *The Cigarette*, was discovered in Remington’s studio after his death. In this painting, four cowboys relax around a small outside a cabin. A plume of smoke rises toward the clear blue-green night sky flecked with a few stars, past a large skin hanging on the side of the cabin. The cabin does not overwhelm the painting but details such as the shadow under the roofline, the seams between logs, the softened edges of the structure, and the tautly stretched skin reveal Remington’s deftness at rendering textures. His use of subdued colors punctuated by the reflected firelight underscores the quiet of the evening’s respite following a long day’s work.

The cowboy in the foreground blocks the campfire so that its muted glow washes out into the middle of the painting. Despite a rifle standing within reach just to the left of the bunkhouse door, this painting does not suggest the foreboding sense of danger characteristic of many of Remington’s other nocturnes. As the evening’s ritual of cigarettes, coffee, and conversation plays out, the cowboy to the left squats and smokes, perhaps telling a story or swapping tales about days gone by.

The lonely vistas and wilderness settings for many of Remington’s works offer, according to Thayer Tolles of the Metropolitan Museum of Art, “a nostalgic, even mythic, look at a rapidly disappearing western frontier, which underwent dramatic transformation in the face of transcontinental transportation, Native American confinement to reservation land, immigration, and industrialization.” The once remote places and outposts yielded to encroaching ranches, farms, and towns, as more people pushed westward each year.

The vast migration and increasing population across the vanishing frontier would also bring the potential for transmission of the pathogens that cause such diseases as tuberculosis, measles, pneumonia, and typhoid. Crossing the plains and deserts by horseback, wagon, or train may have exposed settlers to airborne fungal spores that can cause coccidioidomycosis, commonly called “cocci” or “valley fever.” As cabins, then towns, then cities were built, crowded conditions afforded more opportunities for exposures to pathogens from animal hosts, such as rodents and bats.

Many of Remington’s works portray the Old West as a vast mythical landscape of danger, silence, and beauty. “*The Cigarette*,“ however, captures a quiet moment around a campfire in the dwindling evening light, serving as an elegiac farewell to that time and place.
